# Protracted bacterial bronchitis in adults and comparison to bronchiectasis

**DOI:** 10.1183/23120541.00547-2026

**Published:** 2026-10-12

**Authors:** Patrick Senior, Pedro Viegas, Joseph Winterton, Michael R. Loebinger, Ricardo J. Jose, Janet Stowell, Amelia Bercusson, Aran Singanayagam, Anand Shah

**Affiliations:** 1 Centre for Bacterial Resistance Biology, Department of Infectious diseases, Imperial College London, London, UK; 2 Royal Brompton and Harefield Hospitals, Guy's and St Thomas’ NHS Foundation Trust, London, UK; 3 Pulmonology Department, Unidade Local de Saúde de Gaia/Espinho, Vila Nova de Gaia, Portugal; 4 NHLI, Imperial College, London, UK; 5 Centre for Inflammation and Tissue Repair, UCL, London, UK; 6 These authors contributed equally

## Abstract

Protracted bacterial bronchitis (PBB) is a well-described clinical entity in children, where it is a common cause of wet cough [1], but it is less well understood in adults, in whom there has been no published prospective characterisation. In children, various definitions have been developed, with some based on purely clinical criteria and others mandating microbiological culture of respiratory pathogens [2]. A European Respiratory Society position statement in 2017 defined paediatric PBB as requiring all of*:* 1) presence of a continuous chronic (>4 weeks’ duration) wet or productive cough; 2) absence of symptoms or signs suggestive of other causes of wet or productive cough, and 3) resolution of cough following a 2–4-week course of an appropriate oral antibiotic [3].

## Abstract 2

**PBB is associated with considerable morbidity as reflected by similar rates of exacerbation and prophylactic antibiotic use to a bronchiectasis cohort of moderate clinical severity** https://bit.ly/4vl74JI

*To the Editor:*

Protracted bacterial bronchitis (PBB) is a well-described clinical entity in children, where it is a common cause of wet cough [1], but it is less well understood in adults, in whom there has been no published prospective characterisation. In children, various definitions have been developed, with some based on purely clinical criteria and others mandating microbiological culture of respiratory pathogens [2]. A European Respiratory Society position statement in 2017 defined paediatric PBB as requiring all of*:* 1) presence of a continuous chronic (>4 weeks’ duration) wet or productive cough; 2) absence of symptoms or signs suggestive of other causes of wet or productive cough, and 3) resolution of cough following a 2–4-week course of an appropriate oral antibiotic [3]. Typical respiratory pathogens (including *Haemophilus influenzae*, *Streptococcus pneumoniae*, *Klebsiella pneumoniae*, and *Moraxella catarrhalis)* are commonly isolated [4, 5]. The role of dysbiosis in PBB development is still uncertain, but in a paediatric setting, a dysregulated microbiota, with reduced bacterial diversity and an expansion of *Haemophilus* and *Neisseria*, has been found [6].

Clinically, chronic wet or productive cough is a hallmark of both PBB [3] and bronchiectasis [7], while pathophysiologically both are characterised by bacterial infection and neutrophilic inflammation [2, 3, 7, 8]. Biofilm formation has also been implicated in both diseases [9]. Accordingly, in children, the two conditions have been described as entities on a spectrum of suppuration [10], with potential for progression from PBB to bronchiectasis. In one study, 8.1% of a cohort of paediatric PBB developed bronchiectasis over a two-year period [5]*.*

PBB in adults is an understudied area without consensus definitions or guideline-based treatment strategies [11]. Retrospective data has suggested that PBB may be a distinct phenotype of patients with persistent cough [12] and/or frequent infections in the absence of asthma or COPD. In airways diseases, the impact of mucus plugging is increasingly recognised as a determinant of phenotype and progression [13]. It is plausible that persistent mucus secretion in bacterial bronchitis may be an early precursor leading to permanent structural changes in the lung (*e.g.* bronchiectasis) but there is limited data as yet to support this [14]. In established bronchiectasis, the clinical phenotype is well recognised to be influenced by the underlying airway microbiome, and the interdependence of the microbiome and immune-endotype is being increasingly elucidated [15]. Although microbiological evaluation of adults with PBB has been limited thus far, existing published data suggest similarity to findings in a paediatric setting, with a high proportion of *H. influenzae,* albeit with more frequent isolation of *Pseudomonas aeruginosa* [14] and *K. pneumoniae* [12] in the limited adult data.

Given the lack of published adult PBB literature to date, we sought to characterise adult PBB in a tertiary bronchiectasis referral centre and contrast findings to individuals with bronchiectasis.

A retrospective single-centre analysis was performed between October 2023 and December 2024. All individuals within the bronchiectasis service were screened, with ethical approval provided by the NHS Research Ethics Committee (ref. no: 21/HRA/3400). Individuals within the time period who met our definition of PBB (below) were included in the study. Electronic health record data including demographics, microbiology and radiology were collected and analysed. Chronic *P. aeruginosa* was defined as two or more isolates ≥3 months apart.

The inclusion criteria for the adult PBB cohort were: 1) adults over 18 years, 2) chronic or recurrent productive cough, 3) laboratory evidence of bacterial infection in sputum cultures. Exclusion criteria were: 1) evidence of alternative causes of cough (severe asthma (defined by biologic use), COPD, and smoking ≥5 pack-years) and bronchiectasis (on computed tomography imaging). This definition was based upon a previously published adult case definition [14], and differs from the established paediatric definition through the omission of the requirement for resolution after a course of antibiotics due to the limitations of available medical records in this retrospective study. Individuals with PBB were contrasted with a parallel prospective cohort of adults with bronchiectasis (n=89) (NHS REC ref.: 24/NW/0247) with inclusion criteria as: 1) radiologically confirmed bronchiectasis, and 2) at least one exacerbation in the previous year, with exclusion of individuals with cystic fibrosis or primary ciliary dyskinesia. Clinical and lung function parameters were recorded, and blood and sputum samples were obtained. Continuous variables were summarised as mean±sd or median (interquartile range), and categorical variables as counts and percentages. Group comparisons used Student's t-test or Wilcoxon rank-sum for continuous data and Chi-square or Fisher's exact tests for categorical data. A two-sided p<0.05 was considered significant. Missing data were excluded.

83 subjects were identified who fulfilled the inclusion criteria for PBB and the results are summarised in table 1. Individuals with PBB were younger than those with bronchiectasis (48 *versus* 64 years, p<0.001), while there was a similar female predominance in both cohorts (67% *versus* 76%, p=0.256). Many comorbidities, such as gastro-oesophageal reflux disease and rhinosinusitis were similarly prevalent in both cohorts, which may reflect shared clinical features or risk factors between groups and warrants further study. To enable an equivalent comparison (as having at least one exacerbation in the previous year was a prerequisite of entering the bronchiectasis cohort), a subgroup analysis including only individuals with PBB who had exacerbated within the preceding 12 months was performed. Exacerbation rates were comparable (median 2 *versus* 2, p=0.606), suggestive of similar infective burden and susceptibility. In the bronchiectasis cohort, mean Bronchiectasis Health Questionnaire score±sd [16] was 60.1±9.5 and mean Modified Reiff score of radiological severity was 4.6±3.3; these parameters were not available for the PBB cohort. Lung function parameters (forced vital capacity (FVC), forced expiratory volume in 1 s (FEV_1_) and FEV_1_/FVC) were significantly lower in the bronchiectasis cohort (all p<0.05) reflecting increased structural lung disease. Exhaled nitric oxide fraction readings and presence of eosinophilia (≥0.3×10^9^ per litre) were similar in both groups (p=0.995 and p=0.54, respectively), despite a trend towards increased non-severe asthma in the PBB cohort. This finding may represent clinical overlap or misclassification as results of objective testing of asthma were not retrospectively available in a significant proportion of individuals with PBB. There were no significant differences in asthma treatment step between the two groups however (median step 4 *versus* step 3, p=0.132). Furthermore, exclusion of those with a known diagnosis of asthma did not change the significance of results, apart from significantly increased eosinophilia in individuals with bronchiectasis (9% *versus* 34% p=0.016) and increased azithromycin use in individuals with PBB (25.0% *versus* 8.3% p=0.045).

**TABLE 1:** Comparison of demographics, clinical features, comorbidities, lung function, type II inflammation markers, treatment and microbiology

	PBB (n=83)	Bronchiectasis (n=89)	p-value
**Sex, female**	56 (67.47%)	68 (76.40%)	0.256^#^
**Age**	47.96±18.02	63.66±13.64	<0.001^¶^
**Smoking, pack-years**	0 (0)	0 (2)	<0.001^§^
**Exacerbation rate previous year** ^+^	2 (3), (n=51)	2 (2), (n=89)	0.606^§^
**Comorbidities**			
Asthma	39 (46.99%)	29 (32.58%)	0.076^#^
GORD	12 (14.46%)	11 (12.36%)	0.857^#^
Rhinosinusitis	13 (15.66%)	16 (17.98%)	0.84^#^
Connective tissue disease	0 (0%)	7 (7.87%)	0.039^#^
Hypertension	3 (3.61%)	11 (12.36%)	0.069^#^
Arrhythmia	3 (3.61%)	4 (4.49%)	1.0^#^
Diabetes	1 (1.2%)	3 (3.37%)	0.663^#^
Cardiovascular diseases^**ƒ**^	0 (0%)	5 (5.62%)	0.082^#^
Immunodeficiency	7 (8.43%)	2 (2.25%)	0.139^#^
**Lung function**	n=55	n=88	
FVC, % pred	101.72±16.25	91.17±23.51	0.002^¶^
FEV_1_, % pred	86.83±24.91	75.01±26.22	0.007^¶^
FEV_1_/FVC	72.43±13.26	63.46±12.61	<0.001^¶^
**Type II Inflammation markers**			
*F*_ENO_	16 (19.25), (n=30)	17.5 (13), (n=84)	0.995^§^
Eosinophils ≥0.3×10^9^ per L	15/66 (22.73%)	18/62 (29.03%)	0.54^#^
**Prophylactic antibiotic use**			
Any antibiotic	37/74 (50.0%)	36/89 (40.5%)	0.288^#^
Azithromycin	16/74 (21.6%)	12/89 (13.5%)	0.245^#^
Non-azithromycin oral antibiotic	19/74 (25.7%)	16/89 (18.0%)	0.317^#^
Nebulised antibiotic	6/74 (8.1%)	12/89 (13.5%)	0.401^#^
**Asthma treatment, GINA step**			
0	24/82 (29.27%)	36/89 (40.45%)	0.132^§^
1	4/82 (4.88%)	5/89 (5.62%)	
2	4/82 (4.88%)	1/89 (1.12%)	
3	6/82 (7.32%)	12/89 (13.48%)	
4	29/82 (35.37%)	20/89 (22.47%)	
5	15/82 (18.29%)	15/89 (16.85%)	
Median step	4 (4)	3 (4)	
**Bacteria isolated**			
Any bacteria isolated	83/83 (100%)	55/67 (82%)	
*Pseudomonas aeruginosa*	40 (48.2%)	33 (49.3%)	1.0^##^
*Staphylococcus aureus*	35 (42.2%)	9 (13.4%)	<0.001^##^
*Haemophilus influenzae*	24 (28.9%)	15 (22.4%)	0.455^##^
*Moraxella catarrhalis*	12 (14.5%)	0 (0%)	<0.001^##^
*Streptococcus pneumoniae*	10 (12.0%)	3 (4.5%)	0.145^##^
Chronic *Pseudomonas aeruginosa*	16 (19.28%)	25 (34.72%)	0.046^##^

Data are presented as n (%) or n/N (%), median (interquartile range), or mean±sd, unless otherwise stated. PBB: protracted bacterial bronchitis; GORD: gastro-oesophageal reflux disease; FVC: forced vital capacity; FEV_1_: forced expository volume in 1 s; *F*_ENO_: exhaled nitric oxide fraction; GINA: Global Initiative for Asthma. ^#^: Chi-square test (calculated excluding missing data); ^¶^: t-test. ^§^: Wilcoxon rank-sum test; ^+^: only those with at least one exacerbation in the previous year were included, to allow comparison with the bronchiectasis cohort (for whom at least one exacerbation was an inclusion criteria); ^**ƒ**^: ischaemic heart disease, cerebrovascular disease or peripheral vascular disease; ^##^: Fisher's exact test.

Although *P. aeruginosa* was the most frequently isolated organism in both cohorts, there was a significantly increased proportion of individuals chronically colonised with *P. aeruginosa* in individuals with bronchiectasis compared to PBB (19% *versus* 35%, p=0.046); in the non-asthma subgroup, this disparity became non-significant. This disparity between colonisation and less frequent isolation may, in part, reflect the structural changes inherent in bronchiectasis. These data suggest distinct but overlapping microbiological profiles, with significantly higher isolation of *Staphylococcus aureus* and *M. catarrhalis* in the PBB group. There were similar levels of *H. influenzae* in both conditions*,* an organism previously associated with progression to bronchiectasis [5]. Prophylactic antibiotic use was common in both groups (50% PBB *versus* 40.5% bronchiectasis, p=0.288), with similar usage of azithromycin (22% *versus* 14%, p=0.245) and non-macrolide antibiotics (26% *versus* 18%, p=0.317). Use of nebulised antibiotic prophylaxis was non-significantly higher, however, in the bronchiectasis cohort (8% PBB *versus* 14% bronchiectasis, p=0.402), likely reflective of increased *P. aeruginosa* colonisation.

PBB is associated with considerable morbidity, reflected by similar rates of exacerbation and prophylactic antibiotic use to a bronchiectasis cohort of moderate clinical severity, despite better lung function and lower rates of *P. aeruginosa* colonisation. Our study is limited by its retrospective design, its single-centre tertiary referral setting, and the lack of objective testing for asthma in the PBB cohort. However, it is the first to draw direct comparisons between PBB and bronchiectasis.

Further work is required to clearly define PBB in adults and evaluate prevalence alongside pathophysiological drivers and endotypes. Within a paediatric setting, there is an established spectrum ranging from PBB, chronic suppurative lung disease (CSLD), and bronchiectasis [10]. Whether this is similar in an adult setting is not clear and requires further prospective analysis with a need for longitudinal follow-up to determine whether PBB (or a CSLD-like phenotype) may represent an early form of bronchiectasis, with a proportion progressing to radiologically confirmed disease. Such studies would also allow detailed immunological and microbiological characterisation and predictors of progression thereby improving our understanding of pathophysiology and enabling optimal management. Whether PBB, like bronchiectasis, represents an end phenotype driven by multiple aetiologies and pathophysiological drivers is unclear, but is an important question in the present era of precision therapeutics moderating neutrophilic inflammation in bronchiectasis (*i.e.* dipeptidyl peptidase I inhibitors) [17] and mucus hypersecretion in airway disease (*e.g.* dupilumab in asthma [18] and endobronchial cryotherapy in COPD [19]).

## References

[1] Gilchrist FJ, Ali I, Brodlie M, et al. Developing a core outcome set for children with protracted bacterial bronchitis. ERJ Open Res 2020; 6: 00344-2019. doi:10.1183/23120541.00344-2019 PMC7008134 32055628

[2] Marchant JM, Masters IB, Taylor SM, et al. Evaluation and outcome of young children with chronic cough. Chest 2006; 129: 1132–1141. doi:10.1378/chest.129.5.1132 16685002

[3] Kantar A, Chang AB, Shields MD, et al. ERS statement on protracted bacterial bronchitis in children. Eur Respir J 2017; 50: 1602139. doi:10.1183/13993003.02139-2016 28838975

[4] Chen N, Zhang H, Feng Y. Clinical features and pathogen distributions of microbiological-based protracted bacterial bronchitis in children of different ages in Northeast China. Front Pediatr 2023; 11: 1163014. doi:10.3389/fped.2023.1163014 PMC10162439 37152313

[5] Ruffles TJC, Marchant JM, Masters IB, et al. Outcomes of protracted bacterial bronchitis in children: a 5-year prospective cohort study. Respirology 2021; 26: 241–248. doi:10.1111/resp.13950 33045125

[6] Cuthbertson L, Craven V, Bingle L, et al. The impact of persistent bacterial bronchitis on the pulmonary microbiome of children. PLoS ONE 2017; 12: e0190075. doi:10.1371/journal.pone.0190075 PMC5744971 29281698

[7] Johnson E, Long MB, Chalmers JD. Biomarkers in bronchiectasis. Eur Respir Rev 2024; 33: 230234. doi: 10.1183/16000617.0234-2023 PMC11220624 38960612

[8] Wurzel DF, Marchant JM, Clark JE, et al. Wet cough in children: infective and inflammatory characteristics in broncho-alveolar lavage fluid. Pediatr Pulmonol 2014; 49: 561–568. doi:10.1002/ppul.22792 23788413

[9] Marsh RL, Binks MJ, Smith-Vaughan HC, et al. Prevalence and subtyping of biofilms present in bronchoalveolar lavage from children with protracted bacterial bronchitis or non-cystic fibrosis bronchiectasis: a cross-sectional study. Lancet Microbe 2022; 3: e215–e223. doi:10.1016/S2666-5247(21)00300-1 35544075

[10] Chang AB, Marchant JM. Protracted bacterial bronchitis is a precursor for bronchiectasis in children: myth or maxim? Breathe 2019; 15: 167–170. doi: 10.1183/20734735.0178-2019 PMC6717611 31508153

[11] Everard ML, Priftis K, Koumbourlis AC, et al. Time to re-set our thinking about airways disease: lessons from history, the resurgence of chronic bronchitis/PBB and modern concepts in microbiology. Front Pediatr 2024; 12: 1391290. doi:10.3389/fped.2024.1391290 PMC11190372 38910961

[12] Huang L, Lai K, Zhan C, et al. Clinical characteristics of protracted bacterial bronchitis in adults. Heliyon 2023; 9: e12299. doi: 10.1016/j.heliyon.2022.e12299 PMC9900482 36755583

[13] Huang BK, Elicker BM, Henry TS, et al. Persistent mucus plugs in proximal airways are consequential for airflow limitation in asthma. JCI Insight 2024; 9: e174124. doi:10.1172/jci.insight.174124 PMC10967478 38127464

[14] Finch S, Carreto L, Abo-Leyah H, et al. P141 Persistent bacterial bronchitis in adults – a precursor to bronchiectasis? Thorax 2019; 74: Suppl. 2, A167. doi:10.1136/thorax-2019-BTSabstracts2019.284

[15] Choi H, Ryu S, Keir HR, et al. Inflammatory molecular endotypes in bronchiectasis: a European multicenter cohort study. Am J Respir Crit Care Med 2023; 208: 1166–1176. doi:10.1164/rccm.202303-0499OC 37769155

[16] Spinou A, Siegert RJ, Guan W-J, et al. The development and validation of the Bronchiectasis Health Questionnaire. Eur Respir J 2017; 49: 1601532. doi:10.1183/13993003.01532-2016 28495688

[17] Chalmers JD, Burgel PR, Daley CL, et al. Phase 3 trial of the DPP-1 inhibitor brensocatib in bronchiectasis. N Engl J Med 2025; 392: 1569–1581. doi:10.1056/NEJMoa2411664 40267423

[18] Svenningsen S, Kjarsgaard M, Haider E, et al. Effects of dupilumab on mucus plugging and ventilation defects in patients with moderate-to-severe asthma: a randomized, double-blind, placebo-controlled trial. Am J Respir Crit Care Med 2023; 208: 995–997. doi:10.1164/rccm.202306-1102LE 37603097

[19] Orton CM, Tonkin J, Chan LT, et al. Metered cryospray for the treatment of patients with chronic obstructive pulmonary disease with chronic bronchitis – mechanism of action. A multicentre randomised controlled clinical trial. Eur Respir J 2024; 64: Suppl. 68, OA2797. doi:10.1183/13993003.congress-2024.OA2797

